# Lupinalbin A as the most potent estrogen receptor α- and aryl hydrocarbon receptor agonist in *Eriosema laurentii* de Wild. (Leguminosae)

**DOI:** 10.1186/1472-6882-14-294

**Published:** 2014-08-09

**Authors:** Sylvin Benjamin Ateba, Dieudonné Njamen, Svjetlana Medjakovic, Martin Zehl, Hanspeter Kaehlig, Alois Jungbauer, Liselotte Krenn

**Affiliations:** Laboratory of Animal Physiology, Department of Animal Biology and Physiology, Faculty of Science, University of Yaounde I, P.O. Box 812, Yaounde, Cameroon; Department of Biotechnology, University of Natural Resources and Life Sciences Vienna, Muthgasse 18, A-1190 Vienna, Austria; Christian Doppler Laboratory for Receptor Biotechnology, Muthgasse 18, A-1190 Vienna, Austria; Department of Pharmacognosy, University of Vienna, Althanstrasse 14, A-1090 Vienna, Austria; Institut of Organic Chemistry, University of Vienna, Währingerstraße 38, A-1090 Vienna, Austria

**Keywords:** *Eriosema laurentii* de Wild., Yeast transactivation assays, Estrogen receptor α, Aryl hydrocarbon receptor, Genistein, 2′-Hydroxygenistein, Lupinalbin A

## Abstract

**Background:**

*Eriosema laurentii* De Wild. (Leguminosae) is a plant used in Cameroon against infertility and gynecological or menopausal complaints. In our previous report, a methanol extract of its aerial parts was shown to exhibit estrogenic and aryl hydrocarbon receptor agonistic activities *in vitro* and to prevent menopausal symptoms in ovariectomized Wistar rats.

**Methods:**

In order to determine the major estrogen receptor α (ERα) agonists in the extract, an activity-guided fractionation was performed using the ERα yeast screen. To check whether the ERα active fractions/compounds also accounted for the aryl hydrocarbon receptor (AhR) agonistic activity of the crude methanol extract, they were further tested on the AhR yeast screen.

**Results:**

This study led to the identification of 2′-hydroxygenistein, lupinalbin A and genistein as major estrogenic principles of the extract. 2′-hydroxygenistein and lupinalbin A were, for the first time, also shown to possess an AhR agonistic activity, whereas genistein was not active in this assay. In addition, it was possible to deduce structure-activity relationships.

**Conclusions:**

These results suggest that the identified compounds are the major active principles responsible for the estrogenic and AhR agonistic activities of the crude methanol extract of the aerial parts of *Eriosema laurentii.*

## Background

Plants from the Leguminosae family are well known for their medicinal properties and are used for different purposes in connection with women’s reproductive function, bone density, cardiovascular health and cancer prevention. Their therapeutic effects are very often attributed to the estrogen-like phytoconstituents known as phytoestrogens. For instance, the health benefits of soybeans (*Glycine max* L.) and red clover (*Trifolium pratense* L.) on menopause-related health problems have frequently been ascribed to their high isoflavone content [1–3]. Phytoestrogen exposure occurs primarily through dietary intake of foods or food supplements [4] and these compounds display estrogenic properties due to the binding to estrogen receptors in target cells. Beside estrogen receptors, phytoestrogens can also directly or indirectly modulate a wide range of signaling pathways, including the aryl hydrocarbon receptor (AhR) [5, 6]. Although the effect of this receptor on the reproductive physiology is not yet fully understood, studies have reported its involvement in several antiestrogenic activities [7–9].

*Eriosema laurentii* De Wild. (Leguminosae) is widely distributed in West and Central Africa where it is used as traditional remedy and food [10]. In Cameroon, *E. laurentii* preparations are used for the treatment of infertility and various gynecological and menopausal complaints. Our previous study showed that the methanol extract of the aerial parts of *E. laurentii* exhibited agonistic activities at the estrogen receptor α and the aryl hydrocarbon receptor in yeast transactivation assays and prevented menopause-related symptoms induced by ovariectomy in rats [11]. In addition, the safety profile of this extract indicated a broad safety margin following acute and subchronic oral administration [12]. Therefore, in order to identify the chemical constituents responsible for the observed estrogenic and AhR agonistic activities, an activity-guided fractionation was performed using the ERα- and AhR-yeast assays on recombinant *Saccharomyces cerevisiae* strains.

## Methods

### General experimental procedures

Solvents for extraction and fractionation (analytical grade) as well as HPLC-grade acetonitrile (Chromanorm) and methanol (LiChrosolv) were obtained from VWR International (West Chester, Pennsylvania, USA). Glacial acetic acid (Rotichrom) was purchased from Carl Roth (Karlsruhe, Germany). Genistein (HPLC quality) for the dereplication approach was purchased from Sigma-Aldrich (St. Louis, Missouri, USA).

### Plant material

Aerial parts of *E. laurentii* were collected at July 10th, 2010 in Bazou, West Region of Cameroon. The plant was identified and authenticated by Mr. Victor Nana, botanist at the Cameroon National Herbarium, where a voucher specimen has been deposited under the number 24480/SRF/Cam.

### Preparation of the extract and fractionation

**Figure 1:**
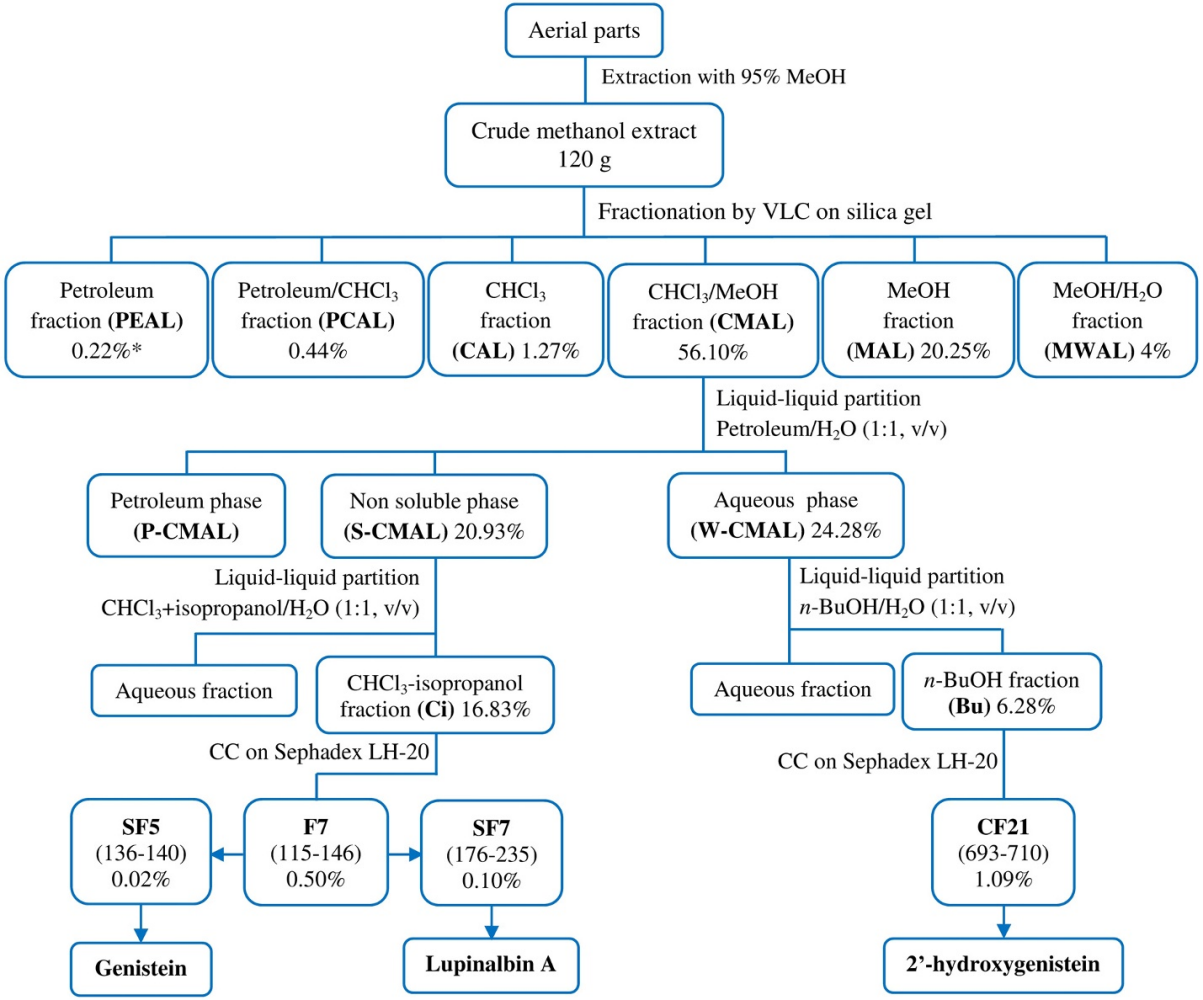
**Scheme of extraction and fractionation of the methanol extract of the aerial parts of** ***Eriosema laurentii.*** * Percent yield on dry weight basis of crude methanol extract.

The air-dried and pulverized aerial parts of *E. laurentii* (2.5 kg) were extracted with 95% methanol at room temperature (3 × 5 L of solvent; 48 h per extraction). The combined solutions were concentrated by evaporation (40°C) to afford 130 g of the methanol extract (=AEL) (Figure 1). The extract (in two portions of 60 g, each) was subjected to vacuum liquid chromatography (column: 5 × 50 cm) on silica gel under successive elution with petroleum ether, petroleum ether-CHCl_3_ (1:1, v/v), CHCl_3_, CHCl_3_-MeOH (1:1, v/v), MeOH and MeOH-H_2_O (7:3, v/v) using 2000 mL of each solvent. The eluates obtained from the two columns with the same solvent were pooled and evaporated to give petroleum ether (=PEAL, 0.26 g), petroleum ether-CHCl_3_ (=PCAL, 0.53 g), CHCl_3_ (=CAL, 1.53 g), CHCl_3_-MeOH (=CMAL, 67.32 g), MeOH (=MAL, 24.3 g) and MeOH-H_2_O (=MWAL, 4.8 g) fractions. Sixty seven grams of the CMAL fraction, which was the only active one in estrogen receptor α yeast assay (yERα), were further fractionated by liquid-liquid partition using petroleum ether-water (1:1,v/v) to afford a water-soluble part (=W-CMAL, 29 g), a petroleum ether-soluble part (=P-CMAL, 11 g) and a non-soluble part (=S-CMAL, 25 g). The most active fraction S-CMAL (22 g) was suspended in CHCl_3_-isopropanol (3:2, v/v) and partitioned with an equal volume of water. The CHCl_3_-isopropanol fraction (=Ci, 17.7 g) was applied to Sephadex LH-20 column chromatography (4 × 50 cm, 15 mL/30 min) and eluted with 80% MeOH to yield eleven fractions (F1-F11) combined according to their TLC profile. F7 (529 mg) was further subjected to a Sephadex LH-20 column chromatography (2 × 50 cm, 5 mL/20 min) and eluted with 70% MeOH to yield 8 subfractions (SF1-SF8). By crystallization, SF7 (108 mg) yielded lupinalbin A (21 mg), whereas genistein was identified in SF5 using a dereplication approach by TLC and HPLC-DAD. The active fraction W-CMAL was partitioned with *n-*BuOH-H_2_O (1:1, v/v) and the *n-*BuOH soluble material (=Bu, 7.5 g) was further chromatographed on Sephadex LH-20 (4 × 50 cm; 10 mL/30 min) and eluted with MeOH-H_2_O mixtures under increasing amounts of MeOH (40% to 80%) to yield 31 combined fractions (CF1-CF31). CF21 (1.3 g) was suspended in EtOAc and the supernatant was evaporated to yield 2′-hydroxygenistein (250 mg).

### Identification of the major active compounds

**Figure 2:**
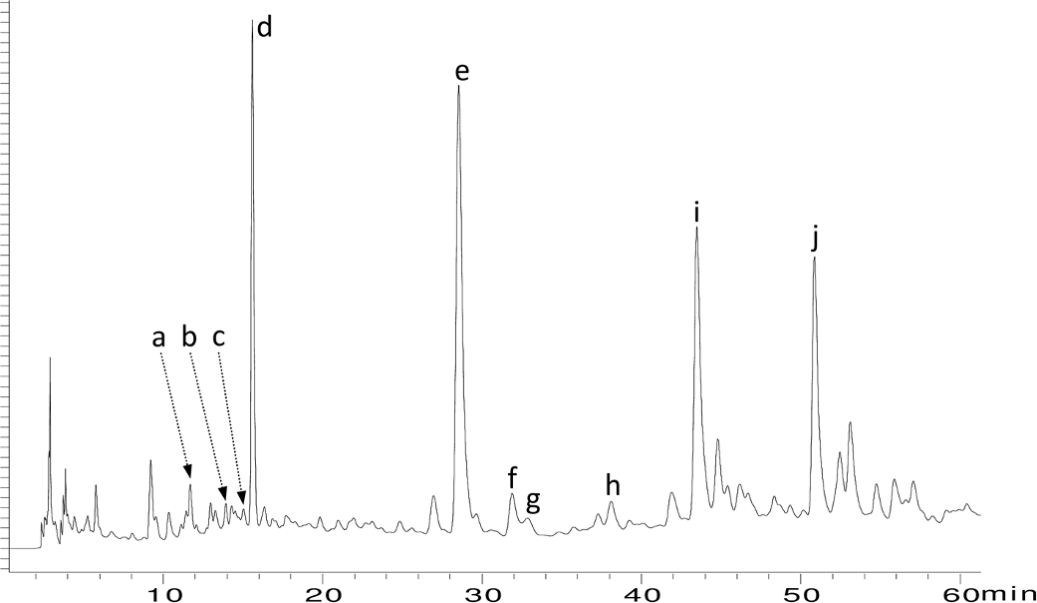
**HPLC-DAD chromatogram (254 nm) of the methanol extract of aerial parts of** ***E. laurentii.*** Peak letters refer to the identified compounds (see Table 2).

The isoflavonoids lupinalbin A and 2′-hydroxygenistein were unambiguously identified by UV, MS, ^1^H and ^13^C NMR; genistein and other components of the extract by TLC-, HPLC- and MS-comparison with authentic material (Figure 2). HPLC-UV analysis was performed on a Shimadzu Prominence HPLC system with a Luna 5μm C18 (2) 100Å (250 × 4.60 mm I.D.) column by a HPLC method previously described [11]. LC-MS was conducted on an UltiMate 3000RSLC-series system (Dionex, Germering, Germany) coupled to an HCT 3D quadrupole ion trap mass spectrometer equipped with an orthogonal ESI source (Bruker Daltonics, Bremen, Germany). HPLC separation in LC-MS experiments was carried out on a Luna 5μm C18 (2) 100Å (250 × 4.60 mm I.D.) column at 25°C using water (adjusted to pH 3.0 with acetic acid) as mobile phase A and acetonitrile/mobile phase A 4:1 (v/v) as mobile phase B. The flow rate was 1.0 mL/min and the following two gradient programs were used for the analysis of selected fractions and isolated compounds:

*Gradient 1*: 5% B (0 min), 5% B (5 min), 20% B (15 min), and 20% B (30 min)

*Gradient 2*: 20% B (0 min), 20% B (5 min), 34% B (10 min), 34% B (30 min), and 36.4% B (37.5 min)

Each gradient was followed by a column cleaning and re-equilibration step. The eluent flow was split in a 1:4 ratio before the ESI ion source, which was operated as follows: capillary voltage: ±3.7 kV, nebulizer: 26 psi (N_2_), dry gas flow: 9 L/min (N_2_), and dry temperature: 340°C. MS^2^, MS^3^, and MS^4^ spectra were obtained in an automated data-dependent acquisition mode (collision gas: He, isolation window: 4 Th, fragmentation amplitude: 1.0 V).

The nuclear magnetic resonance (NMR) data were recorded at 298.2 K on a Bruker Avance DRX 600 instrument operating at 600.13 MHz for ^1^H and 150.91 MHz for ^13^C, respectively. The ^1^H- and ^13^C-NMR data of lupinalbin A and 2′-hydroxygenistein are shown in Table 1 and the MS data for all three compounds are given below. The spectroscopic data were in good correlation to published data (see Table 1) [13, 14].

**Table 1:** ^**1**^ **H- and** ^**13**^ **C-NMR data of lupinalbin A and 2′-hydroxygenistein (in CD**_**3**_ **OD)as compared with literature** [13, 14]

		Lupinalbin A			2′-Hydroxygenistein		
Position		^1^H (ppm) J_H,H_(Hz)	^13^C (ppm)	^13^C (ppm) [13]	^1^H (ppm) J_H,H_(Hz)	^13^C (ppm)	^13^C (ppm) [14]
2	C	—	166.22	166.2	7.866	154.78	155.7
3	C	—	98.65	99.5	—	122.24	120.8
4	C	—	180.06	180.0	—	181.59	180.8
4a	C	—	104.47	104.5	—	104.87	104.8
5	C	—	164.04	164.0	—	161.85	162.3
6	CH	6.281 (d 2.2)	100.92	100.9	6.258 (d 2.2)	99.69	99.3
7	C	—	165.32	165.2	—	164.54	164.7
8	CH	6.483 (d 2.2)	95.83	95.8	6.335 (d 2.2)	94.07	94.0
8a	C	—	156.59	156.6	—	158.04	158.1
1′	C	—	115.23	114.7	—	110.51	109.0
2′	C	—	151.95	151.9	—	158.89	156.8
3′	CH	7.007 (d 2.0)	99.51	98.7	6.424 (d 2.0)	104.87	103.0
4′	C	—	157.81	157.8	—	156.29	159.0
5′	CH	6.903 (dd 2.0/8.5)	114.67	115.2	6.412 (dd 2.0/8.5)	108.31	106.6
6′	CH	7.780 (d 8.5)	122.52	122.5	6.955 (d 8.5)	130.91	132.6

*2′-Hydroxygenistein*: +ESIMS *m/z* 287.0 [M + H]^+^; +ESIMS^2^ (287.0 →) *m/z* 271.0 (8), 268.9 (8), 259.0 (27), 244.9 (35), 230.9 (19), 216.9 (100), 202.9 (11), 189.0 (16), 175.0 (34), 161.0 (23), 153.0 (95), 149.0 (34), 137.0 (11); -ESIMS *m/z* 284.9 [M-H]^-^; -ESIMS^2^ (284.9 →) *m/z* 240.9 (6), 216.9 (100), 198.9 (8), 174.9 (6).

*Lupinalbin A:* +ESIMS *m/z* 285.0 [M + H]^+^; +ESIMS^2^ (285.0 →) *m/z* 256.9 (100), 228.9 (23), 212.9 (10), 185.0 (12), 173.0 (6); +ESIMS^3^ (285.0 → 256.9 →) *m/z* 228.9 (100), 173.2 (7); -ESIMS *m/z* 282.9 [M-H]^-^; -ESIMS^2^ (282.9 →) *m/z* 265.8 (8), 264.8 (17), 254.9 (100), 238.9 (24), 236.9 (7), 226.9 (10), 214.9 (8), 210.9 (8), 172.9 (7); -ESIMS^3^ (282.9 → 254.9 →) *m/z* 236.8 (6), 226.9 (100), 210.9 (14), 182.9 (10).

*Genistein:* +ESIMS *m/z* 271.0 [M + H]^+^; +ESIMS^2^ (271.0 →) *m/z* 253.0 (9), 242.9 (12), 215.0 (25), 153.0 (100), 149.0 (11), 147.1 (37), 145.0 (8).

**Figure 3:**
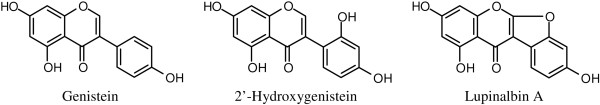
**Chemical structures of 2′-hydroxygenistein, genistein and lupinalbin A.**

The chemical structures of these 3 major compounds are depicted in Figure 3.

For MS identification of other compounds see Table 2.

**Table 2:** **Compounds assigned in the extracts of** ***E. laurentii*** **by HPLC-DAD and HPLC/ESI-MS**

Peak	Rt (min)	UV λ _max(MeOH)_ (nm)	Molecular formula	Identification	Class
**a**	11.89	257sh, 269, 349	C_21_H_20_O_11_	Isoorientin	Flavone
**b**	14.08	270, 337	C_21_H_20_O_10_	Isovitexin	Flavone
**c**	15.09	254, 348	C_21_H_20_O_11_	Luteolin-7-O-glucoside	Flavone
**d**	15.69	260, 325sh	C_21_H_20_O_10_	Genistin	Isoflavone
**e**	28.89	258	C_15_H_10_O_6_	2′-hydroxygenistein	Isoflavone
**f**	32.48	254, 266sh, 349	C_15_H_10_O_6_	Luteolin	Flavone
**g**	33.60	255, 369	C_15_H_10_O_7_	Quercetin	Flavonol
**h**	38.61	255, 357	C_16_H_12_O_7_	Quercetin-3-O-methylether	Flavonol
**i**	43.85	260	C_15_H_10_O_5_	Genistein	Isoflavone
**j**	51.22	256, 281,334	C_15_H_8_O_6_	Lupinalbin A	Coumaronochromone

### Yeast transactivation screens

Disodium hydrogen phosphate dihydrate (Na_2_HPO_4_ · 2H_2_O), potassium chloride (KCl), magnesium sulfate heptahydrate (MgSO_4_ · 7H_2_O), sodium carbonate (Na_2_CO_3_) and ammonium sulfate ((NH_4_)_2_SO_4_) were obtained from Merck (Darmstadt, Germany). Sodium dihydrogen phosphate dihydrate (NaH_2_PO_4_ · 2H_2_O), *N*-lauroylsarcosine (Sarkosyl), *o*-nitrophenol-β-galactopyranoside (ONPG), DL-dithiothreitol (DTT), dimethylsulfoxide (DMSO), D-(+) glucose, estradiol, 5α-dihydroxytestosterone, genistein, progesterone, and β-naphthoflavone were purchased from Sigma-Aldrich (St. Louis, Missouri, USA). Yeast nitrogen base was obtained from Difco (Franklin Lakes, New Jersey, USA), amino acids from Serva Feinbiochemica (Heidelberg, Germany), dropout medium without tryptophan (DO-trp) from Sigma-Aldrich, and dropout supplement media without tryptophan and uracile (CSM-trp-ura) from MP Biomedicals.

LacZ-buffer was composed of 60mM Na_2_HPO_4_ · 2H_2_O, 40mM NaH_2_PO_4_ · 2H_2_O, 10mM KCl, 1mM MgSO_4_ · 7H_2_O, and 1mM DTT. For the *Z-*sarcosyl-buffer, 0.5% *N*-lauroyl-sarcosine was dissolved in LacZ-buffer with 2mM DTT.

The estrogenic and aryl hydrocarbon receptor agonistic activities were assessed using recombinant *Saccharomyces cerevisiae* yeast strains 188R1 and YCM3. The estrogen yeast assay is a two-plasmid system containing an expression plasmid with the human ERα gene and a LacZ reporter plasmid. The construct of the AhR yeast assay contains beside a LacZ reporter plasmid, the human AhR and aryl hydrocarbon receptor nuclear translocator (ARNT) genes integrated in chromosome III. Assay performance and data evaluation have been described previously [5, 15].

Briefly, 1 μL of sample was added in 100 μL of yeast culture (OD_600_ = 0.4) and incubated at 30°C for 5 h and 17 h for ERα and AhR, respectively. After incubation cells were disintegrated by adding 150 μL 2 mM Z-sarcosyl-buffer, the OD_600_ measured and the microtiter plate incubated at 30°C for 20 min for the complete disintegration. In each well, 50 μL of *o*-nitrophenyl β-D-galactopyranoside (4 mg/mL in lacZ-buffer) were added and the plate was incubated at 37°C till the development of yellow color. Afterwards, the reaction was stopped by adding 50 μL 1M Na_2_CO_3,_ the total reaction time noted and the absorption measured at 405 nm (reference wavelength 620 nm).

### Statistical analysis

Data are expressed as means of three independent duplicate experiments ± standard deviation (SD) and analyzed using Student’s *t*-test. Results were considered significant when *p* ≤ 0.05.

## Results and discussion

In order to identify the major compounds responsible for the estrogenic activity of the methanol extract of the aerial parts of *E. laurentii*, an activity-guided fractionation was performed as described in more detail above (Figure 1).

**Figure 4:**
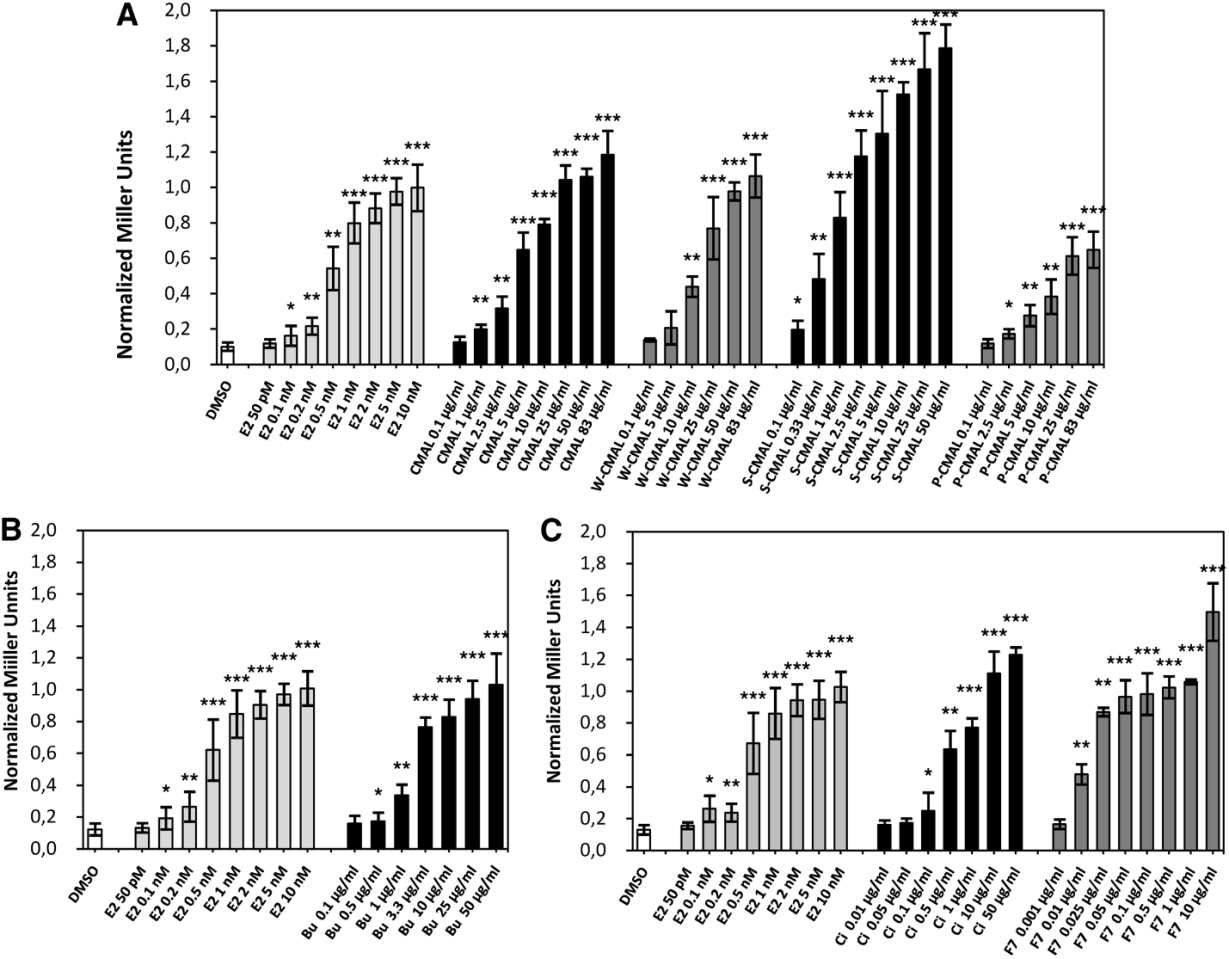
**Normalized Miller Units of CMAL and its sub-fractions in the estrogen receptor α yeast assay.** DMSO: dimethyl sulfoxide, E2: 17β-estradiol, **A)** CMAL: chloroform/methanol fraction of the methanol extract of aerial parts of *Eriosema laurentii*, W-CMAL: aqueous phase of CMAL, S-CMAL: non-soluble part of CMAL, P-CMAL: petroleum ether phase of CMAL, **B)** Bu: *n*-butanol fraction of W-CMAL, **C)** Ci: chloroform-isopropanol fraction of S-CMAL and F7: subfraction 7 of Ci. Data are expressed as mean ± standard deviation of three independent experiments performed in duplicate. *p ≤ 0.05, **p ≤ 0.01 and ***p ≤ 0.001 versus DMSO.

The CHCl_3_-MeOH fraction (CMAL) of the methanol extract from the aerial parts of *E. laurentii* was the only fraction that exhibited estrogenic activity. The three subfractions from CMAL (P-CMAL, S-CMAL and W-CMAL) also induced a dose-dependent and significant β-galactosidase activity in estrogen receptor α yeast assay (yERα) from 1 μg/mL (*p* ≤ 0.05) compared with DMSO (Figure 4A). Fraction S-CMAL showed the strongest effect, and at the dose of 2.5 μg/mL this fraction exhibited a magnitude of response comparable to that of estradiol at the dose of 10 nM. In contrast, the petroleum fraction (P-CMAL) was the weakest one. S-CMAL appeared to be about 10-20 fold more potent than CMAL, whereas the weight of CMAL was only about 2.5-fold the weight of S-CMAL. Plant extracts/fractions contain combinations of different phytochemicals that can interact in a very complex manner leading to antagonistic/synergistic or additive mechanisms. Their fractionation can thus disrupt these molecular interactions resulting in an increase, decrease or abolishment of potency. In such a scenario, the modest potency of CMAL compared to its fraction S-CMAL may be related to the presence of some antagonistic interactions in CMAL. Another reason for the high activity of S-CMAL was the occurrence of genistin in the fraction. This compound was removed by partition in the aqueous fraction (see Figure 1).

Fraction Bu of W-CMAL also induced a β-galactosidase activity in the ERα yeast assay (Figure 4B) and its fractionation yielded 2′-hydroxygenistein (250 mg) as the major component. Fraction Ci of S-CMAL increased the ERα activity in a significant and dose-dependent manner (*p* ≤ 0.05) starting from 0.1 μg/mL. Its magnitude of activity at 1 μg/mL equals that of estradiol at 0.5-1 nM. Moreover, the fraction 7 of Ci (F7) was about a factor of ten more potent than fraction Ci (Figure 4C). From this subfraction, lupinalbin A (21 mg) was isolated. The identification of lupinalbin A and 2′-hydroxygenistein was performed by MS- and one and two-dimensional NMR-experiments. Data showed good correlation with literature (see Table 1). Genistein, one of the major constituents of *E. laurentii* as reported previously [11], was identified using a dereplication approach by TLC, LC-DAD and MS.

**Figure 5:**
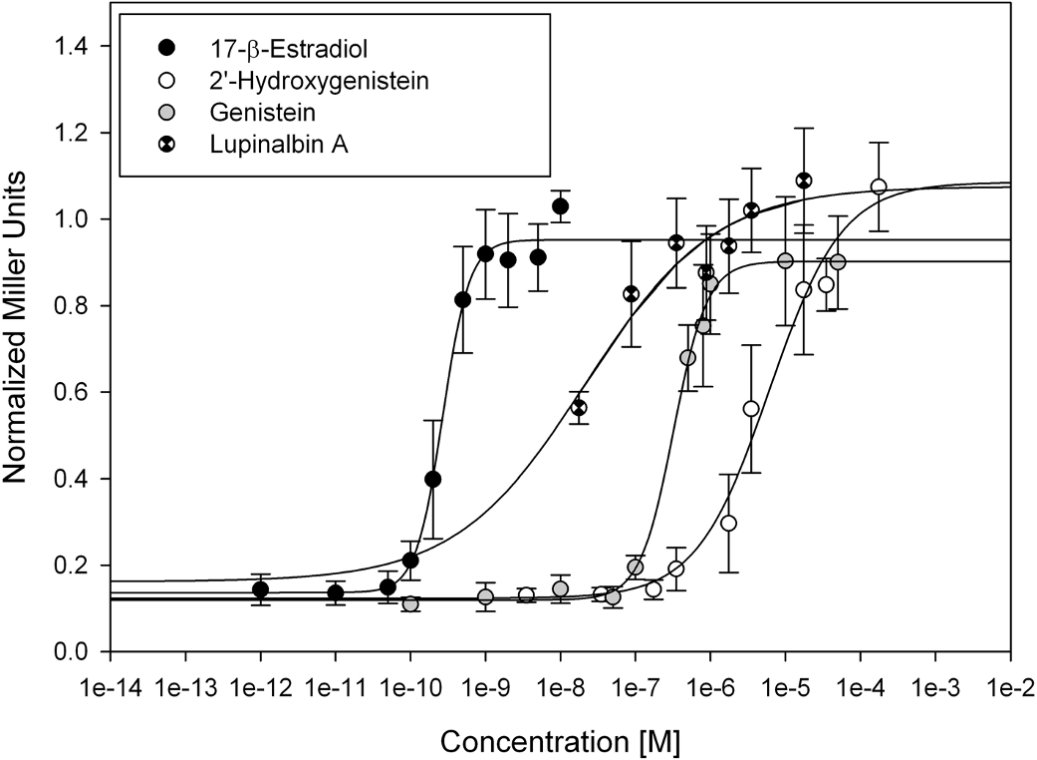
**Dose-response curves of 2′-hydroxygenistein, lupinalbin A and genistein in the estrogen receptor α yeast assay.** Mean values of at least three independent experiments performed in duplicates are shown with standard deviations.

As shown in Figure 5, genistein, 2′-hydroxygenistein and lupinalbin A induced a dose-dependent β-galactosidase activity in the ERα yeast two-hybrid assay. According to the dose-response curves, the EC_50_ values of genistein, 2′-hydroxygenistein and lupinalbin A were 0.32 μM, 6.1 μM and 21.4 nM, respectively, whereas that of 17β-estradiol was 0.27 nM. In line with these results, all three compounds displayed the maximal efficacy of the standard endogenous hormone 17β-estradiol, suggesting that they may be considered as full ERα agonists. On the other hand, it appears that the 2′-hydroxylation of genistein reduced its potency by 19-fold. In contrast, lupinalbin A, a ring-constrained analogue of 2′-hydroxy-genistein was approximately 15-fold more potent than genistein.

**Figure 6:**
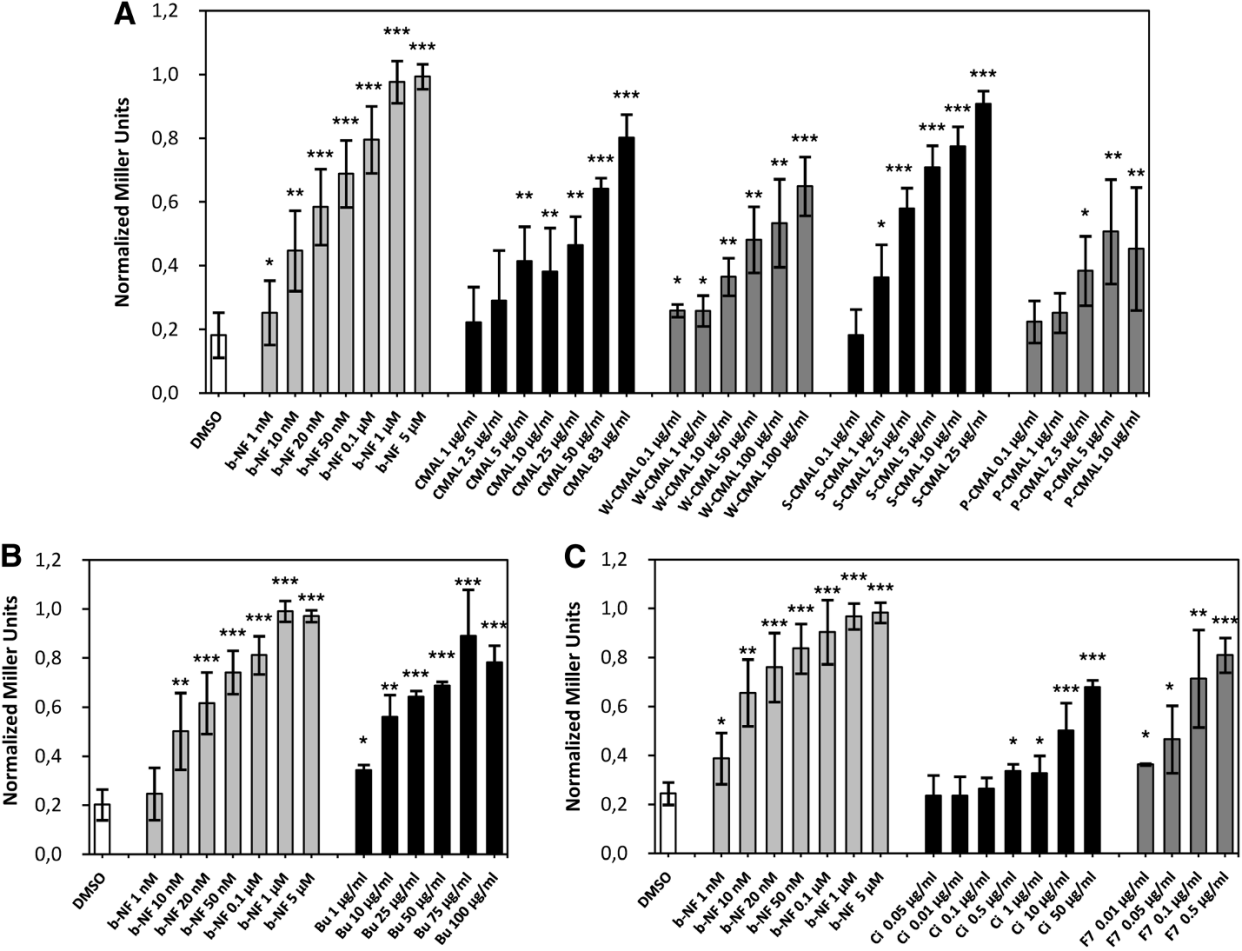
**Normalized Miller Units of CMAL and its sub-fractions in the aryl hydrocarbon receptor yeast assay.** DMSO: dimethyl sulfoxide, b-NF: β-naphthoflavone, **A)** CMAL: chloroform/methanol fraction of the methanol extract of aerial parts of *Eriosema laurentii,* W-CMAL: aqueous phase of CMAL, S-CMAL: non-soluble part of CMAL, P-CMAL: petroleum ether phase of CMAL, **B)** Bu: *n*-butanol fraction of W-CMAL, **C)** Ci: chloroform-isopropanol fraction of S-CMAL and F7: subfraction 7 of Ci. Data are expressed as mean ± standard deviation of three independent experiments performed in duplicate. *p ≤ 0.05, **p ≤ 0.01 and ***p ≤ 0.001 versus DMSO.

In order to check whether the ERα-active fractions/compounds identified in the methanol extract of *E. laurentii* also accounted for the aryl hydrocarbon receptor (AhR) agonistic activity of the crude methanol extract, they were additionally tested on the AhR yeast screen. According to our results, the tested fractions also showed AhR agonistic properties (Figure 6). As in the ERα yeast screen, S-CMAL was the most potent fraction of CMAL (Figure 6A). In comparison to CMAL, it was more potent and activated the AhR already at 2.5 μg/mL in the same manner (*p* ≤ 0.001) as the reference compound β-naphthoflavone at 20 nM (=EC_50_). Fraction Bu (from W-CMAL) as well induced a significant and dose-dependent activity of AhR already starting from 1 μg/mL. Its activity at 10-25 μg/mL equaled that of β-naphtho-flavone at 20 nM (Figure 6B). Similarly, fraction Ci and its sub-fraction F7 induced a notable AhR activity compared with DMSO (Figure 6C). Fraction Ci induced a significant (*p* ≤ 0.05) β-galactosidase activity starting from 0.5 μg/mL. At 10-50 μg/mL, the magnitude of activity of Ci was the same as β-naphthoflavone at its EC_50_-value. Sub-fraction F7 from Ci had the same effect already at 0.05-0.1 μg/mL. A growing body of evidence indicates that AhR/ERα interactions greatly impact the gene expression and proliferative programs of estrogen-sensitive cancers cells. Research addressing this crosstalk showed repressive effects of ligands of AhR on ERα in the estrogen-induced proliferative responses in endometrium and breast cancer cells [16–19]. In respect with this, the activated AhR has been reported to increase the metabolism of E2 via the expression of cytochrome P450 enzymes (CYP1A1 and CYP1B1) [20] and to induce the ubiquitination and proteasomal degradation of ERα in rodent tumors and in ZR-75, T47D, and MCF-7 human breast cancer cells [21, 9]. This degradation of ERα was significantly higher after co-treatment (co-activation) with E2 and 2,3,7,8-tetrachlorodibenzo-*p*-dioxin (TCDD) than after treatment with TCDD alone [9]. AhR can also inhibit ER-dependent transcriptional activities either by binding to the inhibitory xenobiotic response elements (iXRE) located in some ER-dependent promoters (promoter interference) or by the competition for a common pool of co-activators [8]. In addition, knockdown of AhR abrogates the repression of estrogen-dependent gene transcription in MCF-7 breast cancer and ECC-1 endometrial carcinoma cells [22] as well as in the development of colonic tumors in the cecum [23]. According to several reports, these repressive effects of AhR on cancer are mediated by the presence of exogenous ligands of AhR [24, 25]. Our results show that some fractions of *E. laurentii* activated the AhR and thus, active constituents may be considered as exogenous ligands of this receptor. Given these repressive mechanisms of AhR on ERα signaling pathways and due to the coexistence of both receptors in estrogen-sensitive tissues including breast, uterus and ovary our results suggest that the effect of *E. laurentii* on AhR could be of physiologic and anti-tumorigenic relevance. However, besides these anti-estrogenic effects of AhR, data in literature are quite conflicting regarding the implication of AhR in cancer promotion or prevention. It has also been related to cancer initiation or progression via the expression of CYP 450 enzymes (CYP1A1/2 and CYP1B1). The expression of CYP1A1 is part of negative feedback mechanisms regulating the AhR; by metabolization and agonist depletion, CYP1A1 prevents a constitutive activation of the AhR pathway. If this mechanism is impaired the AhR pathway is dysfunctional and toxic responses are the consequence [26]. Uno et al. observed in CYP1A1 knockout mice that although benzo[a]pyrene is metabolized by CYP1A1 to a carcinogenic epoxid form, in the absence of CYP1A1 acute benzo[a]pyrene toxicity is more fatal [27]. Nowadays, CYP1A1 is known to be predominantly important for detoxication of carcinogens and metabolic activation of dietary compounds with cancer preventive activity [28, 29] whereas CYP1B1 is implicated in the metabolic activation of procarcinogens causing DNA adducts formation [30]. In respect with this, up-regulation of CYP1A1 *vs.* CYP1B1 (detoxication/procarcinogen activation) appears to be an indispensable precondition for tumor prevention. However, the underlying mechanisms that modulate this balance still remain unclear depending on the AhR agonists, cell context as well as co-regulators present in different cell types. Therefore, although several constituents of *E. laurentii* are AhR agonists, a final conclusion about their contribution to cancer prevention or progression is not possible, yet.

**Figure 7:**
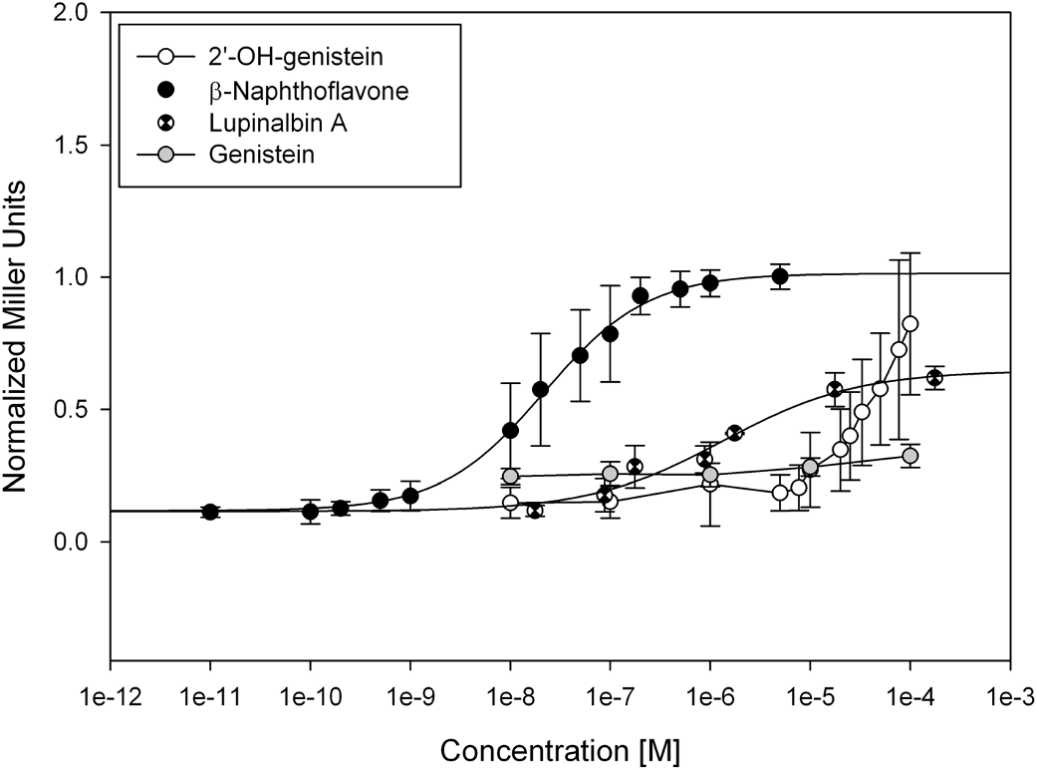
**Dose-response curves of 2′-hydroxygenistein, lupinalbin A and genistein in the aryl hydrocarbon receptor yeast assay.** Mean values of at least three independent experiments performed in duplicates are shown with standard deviations.

As far as the isolated compounds are concerned, 2′-hydroxygenistein and lupinalbin A were found to be active in the AhR yeast assay (Figure 7). The EC_50_ value of lupinalbin A was 1.34 μM while the one of 2′-hydroxygenistein was not determined because a saturation of the receptor was not achieved. To the best of our knowledge, these compounds have not been previously associated with any AhR activity. According to our results, genistein was not active, which is in agreement to previous data obtained with the same yeast system by Medjakovic and Jungbauer [5]. Thus, it appears that hydroxylation at C-2′ of genistein as well as constraining ring B to the benzopyran core by an oxygen bridge are responsible for the observed AhR agonistic activity. Given the maximal efficacy, these compounds may be considered as partial agonists of AhR.

## Conclusions

The activity-guided fractionation of the crude methanol extract of aerial parts of *Eriosema laurentii* lead to the identification of components with estrogenic and aryl hydrocarbon receptor agonistic activities. Genistein, 2′-hydroxygenistein and lupinalbin A, which were identified in or isolated from the most active fractions, were found to be full agonists of ERα. Furthermore, we provide the first report of an agonistic activity of lupinalbin A and 2′-hydroxygenistein towards the AhR, whereas genistein remained without effect at the AhR. The results enabled the deduction of structure-activity relationships for AhR agonistic activity between these compounds for the first time as well. Thus, the data presented in this study confirmed that these phytoconstituents contribute to the estrogenic and aryl hydrocarbon receptor agonistic activities of the extract of *Eriosema laurentii* and might partly account to the beneficial effects of the methanol extract in vivo.

## Abbreviations

AhR: Arylhydrocarbon receptor
b-NF: β-naphthoflavone
E2: 17β-estradiol
ERα: Estrogen receptor alpha.
